# Clinicians’ view on the management of oral health in Parkinson’s disease patients: a qualitative study

**DOI:** 10.1038/s41405-023-00144-w

**Published:** 2023-05-12

**Authors:** Merel C. Verhoeff, Magdalini Thymi, Arnoud N. Brandwijk, Mark S. Heres, Michail Koutris, Henk W. Berendse, Karin D. van Dijk, Frank Lobbezoo

**Affiliations:** 1 Department of Orofacial Pain and Dysfunction, Academic Centre for Dentistry Amsterdam (ACTA), University of Amsterdam and Vrije Universiteit Amsterdam, Amsterdam, The Netherlands; 2 Amsterdam University Medical Centres (Amsterdam UMC), Vrije Universiteit Amsterdam, Neurology, Amsterdam Neuroscience, Amsterdam, The Netherlands; 3 Sleep Wake Centre, Stichting Epilepsie Instellingen Nederland (SEIN), Heemstede, The Netherlands

**Keywords:** Gerodontics, Dental public health, Dentistry

## Abstract

**Background:**

due to numerous motor and non-motor symptoms, dental treatment in patients with Parkinson’s Disease (PD) can be challenging. Knowledge regarding optimal management of oral health in PD patients is lacking.

**Aim:**

to gain a deeper understanding of the experiences of dentists regarding oral health care for PD patients in the Netherlands.

**Material and method:**

semi-structured interviews were conducted with (specialized) dentists working with PD patients. A thematic analysis was performed using a framework-based approach.

**Results:**

ten dentists were interviewed. They reported that dental care in PD patients requires 1) adaptation of timing and length of treatments and consultations, and 2) intensifying preventive measures. Dentists experienced the organization as bureaucratic and difficult. Moreover, differences between being institutionalized or living at home were present. Education and research are needed to improve PD patients’ oral health. The experience level and affinity for treating PD patients positively influences confidence levels of the practitioner. Finally, points of improvement were suggested.

**Conclusion:**

managing oral health in PD patients is challenging, and interdisciplinary collaboration is needed to overcome difficulties. Reducing the bureaucratic burden and improving knowledge could help and stimulate oral health care providers to treat PD patients more effectively and, consequently, improve their oral health.

## Introduction

Parkinson’s Disease (PD) is a neurodegenerative condition that involves the loss of nigral dopaminergic neurons in the brain and widespread accumulation of specific proteins in Lewy bodies [1], leading to non-motor symptoms (e.g., depression, pain, cognitive dysfunction) and motor symptoms (e.g., bradykinesia, tremor, freezing of gait) [2]. PD affects 1–4% of individuals older than 60 years of age, and the incidence increases with ageing [3]. To suppress the symptoms related to PD, patients use many types of medication (e.g., dopaminergic medication, serotonin reuptake inhibitors, and specific anti-psychotics) [4].

During the past few decades, socioeconomic developments and new treatment options in dentistry have resulted in a decreasing number of edentulous patients. As a result, many older persons keep their teeth until late in life [5]. Individuals with PD are known to experience oral health problems, such as xerostomia or sialorrhea, a burning mouth, denture problems, periodontal disease, caries, and pain [6, 7]. Compared to healthy controls, PD patients have a lower number of teeth, an increased amount of dental plaque, caries, and periodontal conditions, problems with chewing and swallowing, and issues with their dentures [6, 7]. Altogether, these examples of worse oral health may result in social distancing and deterioration of their Oral Health-Related Quality of Life (OHRQoL) [8–10].

In The Netherlands, oral-health-related protocols for preventive strategies and dental treatments for institutionalized older individuals have recently been developed [11]. However, in practice, not every nursing home has sufficient staff to comply with these protocols [11]. Moreover, not every frail older individual is institutionalized [12]. Therefore, general dentists will often need to provide care to these non-institutionalized frail older persons, among whom PD patients. Due to their numerous symptoms, the provision of adequate oral health care to PD patients can be challenging. Moreover, a multidisciplinary approach may be required because of the complexity of the disease and the different phenotypes that can be distinguished within the PD-patient population. However, working together with other specialties can be a challenge, especially for dentists working in a general dental practice, due to, for example, barriers in interdisciplinary communication. In addition, some general dentists may lack affinity for geriatric dentistry, which may influence the extent to which they involve themselves in the provision of care for patients with PD. These challenges can lead to insufficiencies in the oral healthcare that PD patients currently receive. Furthermore, it is expected that the prevalence of PD will increase in the near future due to, for example, the ageing of the population [3]. Thus, it is plausible that more dentists will become involved in providing oral health care to older patients in general, including a greater number of patients with PD. Clearly, a properly structured healthcare system is needed to facilitate the delivery of adequate oral health care for PD patients, now and in the future. However, there are still many things unknown about the best possible organization and content of this system. To improve the oral health care of this special-needs population, the input of dental clinicians may provide valuable insights.

This study aims to gain a deeper understanding of the experiences of general and specialized dentists regarding treatment (viz., measures and prevention), organization (viz., politics; rules and regulations; accessibility; existing initiatives for, and points of improvement), and education and research, including competence, of oral health care concerning patients with PD, in order to facilitate the development of a future standardized protocol to structure the oral healthcare system for PD patients, and to improve the current educational and research programs.

## Material and methods

### Design

This study has a qualitative design in the form of semi-structured interviews. On the one hand, semi-structured interviews allow acquiring information regarding the interviewees’ perspectives on pre-assessed topics; on the other hand, they allow collecting information regarding new topics brought up through the conversation. This approach gives methodological flexibility and yields more in-depth knowledge than quantitative research, such as surveys [13].

### Population and eligibility

Purposive sampling was used to select participants, based on specific criteria [14]. The eligibility criteria were as follows: (i) general dentists or specialized dentists working with PD patients, in the Netherlands; (ii) with ≥2 years of working experience; and (iii) who treat or treated (i.e., no longer than two years ago) patients with PD. Dentists with personal or professional affiliations with the interviewer were excluded. Dentists were approached through the personal networks of staff working at the Department of Orofacial Pain and Dysfunction of the Academic Centre for Dentistry Amsterdam (ACTA). Dentists who specialized in geriatric dentistry were approached through the Dutch Association of Gerodontology (viz., Nederlandse Vereniging voor Gerodontologie). Moreover, advertisement took place through social media.

From dentists willing to participate, written informed consent was obtained. This study was approved by the Ethics Committee of ACTA (Reference number 2021-33650).

### Interviews

An Interview topic guide was designed as an agenda to ensure that a systematic collection of information could be assembled. This topic guide consisted of six main themes that reflected our aims (Table 1). Before the study, A.B. and M.H. were trained by M.T., a dentist specialized in orofacial pain and dysfunction and researcher experienced in the conduct of qualitative research. In addition, two pilot interviews were performed to gain more experience before interviewing the participants.

**Table 1:** The main domains of the topic guide used to semi-structure the interviews.

Main domains	
1. Experience	The experience as a general practitioner and/or specialized dentist treating PD patients. (e.g., years of experience; how many patients with PD do you treat?; in what kind of setting do you treat PD patients?)
2. Diagnostics, treatment, and prevention	The interviewee’s view on the diagnostics, prevention, and treatment planning in patients with PD. (e.g., which considerations do you make (regarding diagnostics, treatment and prevention) in patients with PD?; how do you experience interdisciplinary collaboration?)
3. Education	The interviewee’s view on their received education regarding the management of oral health in PD patients, including dentistry, specialization, and refresher courses (e.g., what were your considerations to specialize or not specialize in geriatric dentistry?; what was your experience during your received education?; what are points of improvement in your opinion?)
4. Competence	The interviewee’s feeling of competence regarding the management of oral health in PD patients (e.g., how do you feel about treating PD patients?; what do you think is necessary to improve confidence regarding the treatment of PD patients?)
5. Care system and politics	The interviewee’s view on the current regulations and political aspects concerning oral health in PD patients (e.g., based on your opinion, what are positive aspects of the current care system and politics regarding oral health in PD patients?; do you want to see aspects differently, and if so, why and how?)
6. Professional practice	The interviewee’s view on their professional practice and their view on future improvements regarding oral health in PD patients (e.g., how do you see interdisciplinary collaboration in oral health care in PD patients?; what do you need to experience enough satisfaction in your professional practice?)

*PD* Parkinson’s Disease.

Each interview took place at a date and location chosen by the participants. Prior to the interview, participants filled out a questionnaire with questions on their demographics and education (viz., year of birth, gender, year of graduation, place of graduation, working environment, and years of experience as a specialized dentist) (Table 2). A.B. and M.H. interviewed all participants and audio-recorded the conversations. The interview duration was ~45 min. After that, the recordings were transcribed verbatim, with any information removed that could reveal the interviewee’s identity. After being transcribed, thematic analysis of each interview took place.

**Table 2:** Characteristics of the interviewees.

Participants (*n* = 10)		
Gender [*n* (%)]	Male	2 (20%)
Age [M,SD (range)]		46.2 ± 12.8 (27–64)
Specialized dentist [*n* (%)]		9 (90%)
Experience as a general dentist in years [M, SD (range)]		21.4 ± 12.2 (3–39)
Experience as a specialized dentist in years [M, SD (range)]		9.9 ± 5.4 (1–15)
Graduation type^a^ [*n* (%)]	Portfolio	4 (40%)
	Post-initial education	5 (50%)

*n* numbers, *%* percentage, *M* Mean, *SD* Standard deviation.

^a^In the Netherlands, two types of educational programs can be chosen to specialize in geriatric dentistry: “portfolio” or “post-initial educational program”(see results, section education).

### Analysis

Thematic analysis of acquired data was performed using a framework to identify emerging themes and concepts [13]. The analysis was carried out in the following steps: (i) each transcript was investigated for the identification of initial themes by A.B. and M.H.; (ii) conceptually related initial themes from the available interviews were grouped into main themes, each of which consisting of subthemes, by A.B., M.H., and M.V.; (iii) a thematic chart was created, in which the first column represented the main themes, under which each subtheme was presented in the second column by A.B., M.H., and M.V.; and (iv) the synthesis of the data and formulation of conclusions per subtheme, and subsequently per main theme, took place by A.B., M.H., M.V., and M.T. When new initial themes arose from the interviews, or more knowledge regarding the data led to new insights that required a different categorization, stepping backwards in the analytical process was allowed. Moreover, interviews were performed until no new themes emerged. To confirm this saturation, two more interviews were performed. For the analysis, a software program “ATLAS.ti” (Scientific Software Development GmbH, Berlin, Germany) was used to analyze the data and synthesize the results. A thematic chart with the summary of the results was created in Microsoft Excel software (Microsoft Corporation, Redmond, Washington, U.S.) (Table 3). Transcripts were not returned to the interviewees for comments or corrections, and no interviews were repeated. Although this article focuses on PD patients, some results may also be applicable to a broader population of frail older individuals.

**Table 3:** Main themes, subthemes, and summary of the clinicians’ vision regarding these (sub-)themes.

Main themes	Subthemes	Summary
1. Treatment	Measures	- Extended treatment time to give “rest, relaxation, and predictability”. This reduces stress and anxiety during the treatment and thereby reduces the severity of the tremor.
		- Moment of treatment: tailored to the patient, based on medication intake and the wearing-off symptoms.
		- Medication (and sedation) could be considered in some situations to improve the feasibility of the treatment.
		- Material use: adjust your choice of materials when necessary (e.g., glass-ionomer instead of composite, direct restorations)
		- Treatment planning should be based on PD patients’ health situation and prospects.
		- Preventive measures like shortened arch or treatment such as implants should be considered.
		- Difficulties concerning wearing a prosthesis are frequently present in patients with PD (e.g., dry mouth and hypersalivation due to polypharmacy, motor problems)
		- Difficulties concerning self-care is frequently present in patients with PD.
		- Treatment location is adjusted when necessary (e.g., ground floor, wide corridors, hoist availability, extra pillows).
		- Treatment at home is limited; only preventive measures and simple restorations can be performed. However, it can also contribute to a good assessment of a patient’s living situation. Besides, it could be beneficial to understand and memorize the instructions.
		- Interdisciplinary collaboration, both within and outside dentistry, is needed to result in a better quality of care for PD patients.
		- GP’s and SD’s should be flexible (e.g., treatment environment, material use, difficult predictability of severity of the disease).
	Prevention	- If PD patients are at risk, preventive measures should be taken to reduce the risk of oral diseases (e.g., reducing the interval of oral hygiene measures and monitoring; fluoride application; 5000ppm fluoride toothpaste).
		- Caregivers could be included in the daily care when self-care is no longer feasible for the PD patient themself.
2. Organization	Politics, rules and regulations	- Oral health care for older individuals, let alone PD patients, is not scheduled on the political agenda.
		- Differences between PD patients living at home and institutionalized patients are becoming larger (e.g., financial support)
		- GP and SD are not supported when practicing dentistry in general offices; however, they are well supported with the administrational burden when practicing in special centers.
		- Information on support systems for PD patients living at home is lacking.
		- interdisciplinary work is recommended (e.g., standard referral to a dentist when PD diagnosis is made; intensifying contact between (oral) health care providers around the PD patient).
		- Nursing homes implement structural improvements to ensure better oral health care internally (e.g., “oral health care coordinator” or a GP or SD working in nursing homes).
		- Protocolized standard care is lacking in oral health care of PD patients.
		- Oral care is not included in the inspection of nursing homes; when doing so, oral health care could be improved.
	Accessibility	- Patients’ mobility is failing (e.g., to physically come to the general office; location itself because of stairs or parking).
		- Possibilities to receive special care is unclear (e.g., which dentists are competent in treating PD patients; finance).
		- PD patients are dependent on caregivers or family members.
		- PD patients already experience an overload of (para-)medic support.
		- Postpone treatment (e.g., oral health is subordinate to other PD-related problems; no oral health problems, no visit necessary; cognitive problems regarding organization).
		- The experience of SD is that PD patients prefer to stay with their dentists as long as possible.
	Existing initiatives for improvement	- Currently, dentists are included in a Dutch initiative called “Parkinson Net”, which establishes a network of (para-)medic health care providers, to improve, for example, interdisciplinary collaboration, better accessibility for PD patients to (para-)medic health care providers, and enlarge expertise of (para-)medic health care providers.
		- Commercial companies are drawing attention to the need for (oral) health care in special needs groups. This could improve health care organizations in, for example, institutions. However, (oral) health care practitioners find it difficult to work along because of the motive of profit-seeking.
	Points of improvement	- SD urge the GPs: “When patients are not coming to the general office, call them yourself!”
		- Introducing a “vulnerability score” gives insight into the need and the level of compensation for oral health care in PD patients, including those living at home.
		- Protocol regarding oral health care in PD patients (including institutionalized PD patients and PD patients living at home).
		- Establishing better conveyance to dental and medical clinics could lower the barrier for PD patients and caregivers.
3. Education and research	Competence	- The GP lacks knowledge regarding oral health in older individuals, especially in PD patients (e.g., practical skills; paramedic therapies like speech therapy; salivary problems; medical diseases; best referral moment; and medication usage).
		- The affinity of GP regarding geriatric dentistry is associated with their knowledge level.
		- When experience is lacking, the SD experience confidence problems (e.g., knowledge; practical skills; frequency of dental visits).
		- Despite the experience of the SD, communication with PD patients when cognitive decline is present is difficult.
		- Working interdisciplinary as a SD helps overcome confidence problems and difficulties when no guideline for treating PD patients is present.
	Education	- Recently, special needs groups have been integrated into the dentistry curriculum or in the post-initial program. However, in the past, this was lacking.
		- Both post-initial programs or the alternative by means of the “portfolio system” allows becoming a SD. Both paths are giving the SD more confidence in treating PD patients.
		- Reasons to start the post-initial program to become a SD are: (i) the care for special needs groups is challenging; (ii) desire to learn more regarding general medicine; and (iii) affinity with geriatric dentistry.
		- Reasons to not start the post-initial program to become a SD are (i) significant investment of time; and (ii) physically demanding work.
		- Current post-initial programs are renewed to ensure quality.
	Research	- Detailed research regarding the etiology of worse oral health in PD patients is needed (e.g., gut flora; oral bacteria; dry mouth; oral hygiene; ageing in general).
		- Detailed research regarding treatment options for PD patients is needed (e.g., shortened dental arch; implants).
		- Research on the PD patients’ and other health care providers’ views on oral health in PD patients is needed.

*GP* General Practitioner, *SD* Specialized dentists.

## Results

All interviews took place between October 2021 and March 2022. After interviewing eight of the twelve dentists that agreed to participate, saturation was achieved. Two more interviews were conducted, which confirmed that no new themes were brought up. Thus, full saturation was obtained and ten interviewees were included in the analyses (Table 2). Below, an overview of the results for each main theme and the respective sub-themes is presented, supported by some quotes of the participants (in *italics*). The summary of the results can be found in Table 3.

### Treatment

#### Measures

The participants suggested that appropriate measures should be taken to ensure that the treatment for PD patients is feasible (Table 3). Oral health care providers should be flexible to ensure that patients with PD are as comfortable as possible to reduce the chance of difficulties during treatment (e.g., tremors). Measures that could be implemented are, for example, extended treatment time, or planning the treatment in the timeframe when patients experience the most benefit out of their dopaminergic medication. Furthermore, it should be noted that problems with dentures can occur due to dry mouth or motor problems. Finally, oral health care providers should consider treatment options like a shortened dental arch or dental implants for the (functional) rehabilitation of the masticatory system: *“PD patients who lean forward have a rigid mask face, where the musculature is completely smoothed out, and saliva flows a bit from the mouth, a lower denture also slides out of that mouth. A PD patient would then benefit from 2 small implants to give more retention to the prosthesis. It is not that the least invasive care is always chosen. It is looking at what the demand for care and need for care is.”* (Table 3).

#### Prevention

Preventive strategies are important when the oral health of patients with PD is at risk (Table 3). Not only are conventional preventive measures appropriate (e.g., 5000 ppm fluoride, and increased frequency of dental visits) but also someone’s social support system could play an important role (e.g., taking over self-care): “*It would be nice if they could get a little assistance with self-care. I think these things are very important for patients with PD. Respecting what is possible and interfering on time when it is no longer possible.”* (Table 3).

### Organization

#### Politics, rules, and regulations

Oral health care in older individuals is not an item on the political agenda (Table 3). Moreover, differences exist between oral health care for institutionalized older individuals with PD and patients living at home. For example, oral health care providers who treat PD patients are well supported when working in institutions. However, when working in the general dental office, it is difficult to get the right help and financial support for PD patients. Besides, when PD patients live at home, this can result in fragmented care in which many support systems are involved: “*Home care is extremely fragmented in the Netherlands. In a tiny province, where I live, there are already 36 home care organizations. Often it is just one self-employed person, but these 36 home care organizations are all managed differently.”* Information about these support systems is lacking (e.g., entitlement of getting the right help; which institutions exists); thus, getting the care that PD patients need is difficult. Moreover, protocolized standard care is lacking in oral health care for PD patients. Finally, interdisciplinary collaboration is recommended. For example, one of the participants suggested that *“neurologists have to refer PD patients to their general dentist when the PD diagnosis is established”*; this to screen for possible oral health-related problems and to immediately start preventive strategies to ensure an as good as possible oral health. Besides, all participants expressed the need of intensified contact between all (oral) health care providers around the PD patient (Table 3).

#### Accessibility

Especially patients with PD who live at home experience difficulties receiving the care they need: *“It is a challenge to reach PD patients at home. It can be overwhelming for them, as there are usually many caregivers surrounding PD patients.”* Indirectly, this can be due to regulations and politics. However, directly, the accessibility of oral care for PD patients is lacking (e.g., failing mobility, dependence on caregivers or family members) (Table 3). In addition, it is often unclear what the possibilities are for receiving oral health care that PD patients need (e.g., finding dentists who are willing to treat PD patients). Therefore, PD patients are often postponing treatment. Moreover, oral health is often found to be subordinate to PD-related symptoms: the interviewees often hear patients say they believe that when no problems occur, no dental visit is necessary (“*Because PD patients have many different care aspects, the oral health can easily be overlooked.”)* (Table 3).

#### Existing initiatives for improvement

Some national initiatives were started to improve care for PD patients (Table 3). One example is “ParkinsonNet”, which establishes a network of (para-)medic health care providers. These existing initiatives focus on interdisciplinary treatment, better accessibility for PD patients, and enlarging the expertise of (para-)medical health care providers. Furthermore, commercial companies, by way of their modus operandi in the market, contribute to increased attention to the need for (oral) health care in special needs groups. Although (oral) health care practitioners find it difficult to work with such companies, (*“I sometimes have doubts, because I think that there is a very special business model behind it.”)* they improve the visibility of problems in our society (Table 3).

#### Points of improvement

The participants suggested some practical points to improve dental care for PD patients (Table 3). Examples are the development of protocol-based oral health care, establishing better conveyance to dental and medical clinics, and introducing a “vulnerability score” that gives insight into the need and the level of financial compensation for oral health care in PD patients (including PD patients living at home) (Table 3).

### Education and research

#### Competence

Encountering difficulties in treating patients with PD is present in both general practitioners and specialized dentists (Table 3). Although the specialized dentists see that general practitioners lack knowledge regarding oral health in older individuals, and especially in PD patients, they also experience less confidence themselves in understanding PD as a disease and in treating PD patients (“*Often I felt: If I had learned this during my regular training, I would have understood it better.”)*. Working interdisciplinary as a specialized dentist is considered to help overcome such uncertainties (*“[..] Together with medical students, we wrote a kind of plan for someone. So that’s how you learn to work together in a multidisciplinary way, which I think is extremely important for the future. That you know how to contact each other. And you learn how to visit an older person [..].”)* (Table 3).

#### Education

In the Netherlands, the Dutch Association of Gerodontology recognizes dentists as specialized dentists when a portfolio has been submitted that meets specific requirements. Also, a three-year post-graduate program exists, however, participation is not obligatory to meet the requirements, and a portfolio has to be submitted all the same. Both options for recognition give dentists more confidence in treating PD patients (Table 3). Reasons to start the post-graduate program are the desire to learn more regarding general medicine related to the older patient, and an affinity with geriatric dentistry (“*It is a wonderful profession, very diverse. You are challenged regarding medical knowledge, from medication to diagnosis [..]. That is what makes the profession so much fun. Technically it is not very challenging, but in other ways, it is very special.”)*. However, dentists experience some barriers to start the post-graduate program, such as the significant time-investment and the physical demands of working as a dentist specializing in geriatrics (“[..] *For example, you have to help them get in and out of their wheelchair, you sometimes stand in an awkward position and sometimes they cannot be positioned far enough backwards.”*) (Table 3).

#### Research

More research is needed to explore the etiology and pathophysiology of reduced oral health in PD patients, and to develop better treatment options (Table 3). In addition, research on the PD patients’ and other health care providers’ views on oral health in PD patients is needed (Table 3).

## Discussion

This study aimed to gain a deeper understanding of the experiences of general and specialized dentists regarding treatment, organization, and education and research in oral health care for patients with PD. The results showed that dentists (viz., general and specialized dentists) are experiencing difficulties in treatment, organization, and education and research when working on oral health in PD patients.

Even though the results of the current study are based on the population of PD patients and on the specific situation in the Netherlands, they are in line with five out of six international recommendations that were recently provided by the Lancet Commission on Oral Health (Table 4):[15] caregivers should be encouraged to participate in oral health care of PD patients (recommendation 1); measures should be taken to improve oral health care for PD patients living at home (recommendations 2 and 4); more research is needed to improve decision making (recommendation 5); and difficulties with reimbursement of oral health in PD patients influence oral health negatively, and should be addressed (recommendations 2 and 6). Besides, a seventh recommendation was recently added by Lobbezoo & Aarab (2021, 2022) to stimulate interdisciplinary collaboration between medicine and dentistry (Table 4) [16, 17]., which was also in line with our current results that the participants of this qualitative research agree that interdisciplinary collaboration is needed (recommendation 7).

**Table 4:** Six key recommendations for the new WHO global strategy for oral health, published by the Lancet Commission on Oral Health (Benzian et al., 2021).

1.	Inclusion and community engagement
2.	Place equity and social justice at the core
3.	Tackle sugars as a major common risk factor
4.	Embrace major system reforms
5.	Better data for decision making
6.	Close financing gaps
7.	Promoting interprofessional collaboration between medical doctors and dentists in research, education, prevention, and care provision.

The seventh recommendation was proposed by Lobbezoo & Aarab (2022).

### Treatment

The results of the present study showed that dentists need to be flexible, and that appropriate (preventive) measures should be taken to ensure that the treatment of PD patients will be feasible.

Over the past decades, losing all one’s teeth has become less and less likely, which provides the (partly) dentate older individual with, amongst others, enhanced chewing ability and a better oral health-related quality of life. However, difficulties could arise when older adults still have their own teeth but cannot sufficiently provide self-care. This could, for example, result in oral inflammation, decay of teeth, and orofacial pain. Unfortunately, not every dental office is geared towards the older population (e.g., logistics, location). This causes specific difficulties in preventing the older individual’s oral health from getting worse. When focusing on the PD patient, the problems may be more extensive (e.g., individual phenotype; dexterity of arms and fingers; dependence on the caregiver). For example, in Denmark, it was found that patients with PD are irregular visitors of the dental office, and that they seek help for problem-solving treatments instead of prevention [18]. When analyzing treatment options, implants may help stabilize, for example, a prosthesis. On the other hand, bruxism, a repetitive jaw-muscle activity characterized by clenching or grinding of the teeth and/or by bracing or thrusting of the mandible”[19], is a risk factor for implant failure [20–22]. Because motor symptoms in PD patients also occur in the orofacial area (e.g., dyskinesia), and bruxism is more prevalent in PD patients than in healthy controls [23], this treatment option could therefore be a risk. However, when difficulties with a prosthesis occur, the resulting reduction in OHRQoL may further worsen the quality of life of the PD patient [24, 25]. In such situations, it is not advisable that these treatment options should be excluded a priori. On the other hand, because of the complexity, it is always advisable to work in an interdisciplinary manner, and to consider, e.g., physiotherapeutic options, although the effectiveness of such options has not yet been fully demonstrated [26, 27].

### Organization

The results of the present study highlight the need for: 1. a reduction of bureaucracy and workload; 2. more clarity about the organizational options for the dentist treating PD patients and for the PD patient itself; and 3. increased interdisciplinary collaboration.

Ageing of the population will be one of the causes of an exponential growth in the prevalence of PD in the near future. Moreover, older adults need intensified regular care to prevent oral health-related problems. However, the number of general dentists in the Netherlands is insufficient, let alone that there are enough dentists with an affinity for treating older adults and PD patients. Annually, the Dutch government only makes 259 training positions available, divided over four universities, for students to become dentists. This while approximately 1300-2075 candidates are applying to acquire a training position each year, indicating that dentistry is not lacking popularity amongst prospective students. It may thus be advisable to increase the number of training positions, because a structural shortage of dentists may jeopardize the life-cycle OHRQoL of PD patients in the Netherlands [28].

Not only the shortage of dentists, and therefore the workload, is jeopardizing good-quality oral health care in PD patients, but also the ever-increasing administrative load [29]. Furthermore, the government encourage older individuals to live as long as possible in their own home [12], although the care for institutionalized older adults is better regulated. Even older individuals with a strong social network are experiencing problems, so the situation for older adults with a low socioeconomic status is getting more and more arduous [30]. Should we, as caregivers, researchers, teachers, and politicians, not steer towards qualitatively better longevity at home?

Moreover, organizations (e.g., hospitals, dental practices, health care institutions) are focusing on the responsibility of the individual, clinical prevention, and health education of the individual - in this context the PD patient. However, these downstream interventions are insufficient for long-lasting changes. A balance should be found between upstream interventions (viz., health policy) and downstream interventions [31]. Watt et al. (2007) already draw the conclusion that psychosocial, economic, environmental, and political determinants should not be underestimated in influencing the oral health of the older individuals [31]. However, it seems like no reorganization in oral health care in older individuals – in this context the PD patient – has taken place yet.

### Education and research

The results of the present research highlight the compelling need for more studies focussing on the etiology of oral conditions and the development of better treatments for PD patients, as well as improved education with less barriers for dentists with affinity for the geriatric population.

Geriatric care, including care for PD patients, is not or only minimally included in the general dental curriculum. Therefore, students are not graduating with competence levels that exceed “learner” or “competent” regarding this topic [32]. The consequence of this could be that students will be (un)consciously incompetent in geriatric dentistry. Moreover, dentistry is one of the longest studies in the Netherlands, and substantial efforts of dental students are required for them to graduate as a dentist. Hence, because of the already large effort required to become a dentist, the threshold to start a specialization is high, even though the student may have affinity for dentistry focusing on geriatrics. Although in the Netherlands it is allowed to treat geriatric patients without a specialization, dentists may not feel competent to do so. Recently, another research among dentists treating people with movement disorders in Texas, United States of America, was published [33]. The authors showed that these dentists are willing to treat patients with PD, but that they would like to receive more specialized education on this topic to feel themselves competent enough to treat this population [33]. How this aspect is organized in other countries and which barriers are experienced there, should be addressed in a future research project.

As mentioned above, when dentists want to differentiate as a specialist in geriatrics, there are two possibilities to do so in the Netherlands. Apart from a formal three-year post-graduate educational program including a portfolio, for dentists with sufficient clinical experience a portfolio to demonstrate their work experience may suffice. Although the first group enrolls in a structured educational program to obtain a comprehensive knowledge level, dentists in the second group often have more clinical experience in this area of interest. While this dualistic system increases educational inequality, it also gives the already small group of people interested in specialization the chance to choose an option that fits their needs and personal circumstances.

Importantly, there is a lack of good-quality research regarding oral health in PD patients. Consequently, practitioners cannot gather sufficient evidence-based information on oral health in PD patients, especially on how to treat them. Moreover, it is only a recent development that researchers are expected to share their findings with the general public (e.g., lectures on media channels). Until now, this was not common, with the consequence that neither general dental practitioners nor the general public were fully aware of the recent developments. Accordingly, the general public is not familiar with, for example, the importance of good and stable oral health. Thus, people will not be (internally) motivated to ask for the oral care they need, and oral health care providers are not stimulated to acquire knowledge based on the questions asked.

To conclude, the educational structure may not encourage specializing in geriatric dentistry, although the need for dentists to treat geriatric patients is increasing. Besides, there is little evidence on managing oral health problems of PD patients. Therefore, it is not likely that general and specialized dentists are able to collect enough evidence-based information to support their clinical decision making.

### Limitations of the study and recommendations for future studies

Some limitations of the present study have to be pointed out. First, of the ten interviewees, only one general dentist was included whereas the others were specialized. Besides, no other oral health care providers than dentists, such as dental hygienists, were approached. Since dental hygienists may see certain patients more frequently than dentists, this could have been a valuable addition. For future research, it is recommended to approach the dental team in a more extensive form. Second, the included group of interviewees is small. However, we followed the guidelines for qualitative research [13, 34] and stopped including interviewees after saturation was achieved. This is a validated and approved approach, and therefore we are confident that our conclusions are valid. Third, we asked the participants to describe their experience as a general dentist and as a specialized dentist. However, during their work in general practice, which usually continues parttime while also working as a specialized dentist, their experience with treating PD patients could have developed further. Therefore, the outcome is probably an underestimation of their experience in this field of expertise.

## Conclusion

The interviewees highlighted deficiencies in the Dutch oral health care system regarding treatment, organization, education and research concerning oral health care for PD patients. Mainly, education is lacking, since this topic is not well represented in the current dental curriculum. In addition, dentists’ knowledge regarding oral health in PD patients is limited. Although less bureaucracy and more interdisciplinary approaches are likely to improve oral health care in PD patients, these issues represent major societal, political, and educational challenges. Furthermore, intensified and higher-quality research regarding oral health in PD patients is needed to close the knowledge gap and to increase the confidence level of general and specialized dentists.

## Data Availability

The participants of this study did not give written consent for their data to be shared publicly, so due to the sensitive nature of the research supporting data is not available.
